# Joint effect of 25-hydroxyvitamin D and secondhand smoke exposure on hypertension in non-smoking women of childbearing age: NHANES 2007-2014

**DOI:** 10.1186/s12940-021-00803-1

**Published:** 2021-11-15

**Authors:** Qianqian Shen, Qian Xu, Guoju Li, Lisheng Ren, Zhenhong Zhang, Yangting Zhang, Zhaoyi Zhong, Xiaona Li, Qiuzhen Wang

**Affiliations:** 1 Public Health School, Institute of Human Nutrition, Medical College of Qingdao University, Gate 2, Haoyuan, Ningde Road, Qingdao, China; 2 Qingdao Women and Children’s Hospital, Qingdao University, Qingdao, China; 3 The Affiliated Hospital of Qingdao University, Qingdao, China

**Keywords:** 25-hydroxyvitamin D, Secondhand smoke, Hypertension, Women of childbearing age, Interaction

## Abstract

**Background:**

Vitamin D deficiency (VDD) may increase the risk of hypertension in women of childbearing age, who may be exposed to secondhand smoke (SHS) simultaneously. Till now, few studies have investigated the joint effects of VDD and SHS on hypertension in this population. We evaluated whether exposure to SHS modified the association between VDD and hypertension.

**Methods:**

Data from National Health and Nutrition Examination Surveys (NHANES) 2007-2014 were analyzed. Our research subjects were 2826 nonsmoking and nonpregnant women of childbearing age (20-44 years old). Hypertension was defined based either on systolic blood pressure (SBP) ≥ 130 mmHg and/or diastolic blood pressure (DBP) ≥ 80 mmHg or on now taking prescribed medicine for hypertension. The directed acyclic graphs (DAG) and the back-door criterion were used to select a minimal sufficient adjustment set of variables (MSAs) that would identify the unconfounded effect of 25(OH)D and hypertension. The interactive effect of VDD and SHS on hypertension was evaluated by using logistic regression models, followed by strata-specific analyses.

**Results:**

The prevalence of VDD in the hypertension group was significantly higher than that in the non-hypertension group (48.2% vs 41.0%, *P* = 0.008), as well as the exposure rate of SHS (39.1% vs 33.8%, *P* = 0.017). VDD was independently associated with nearly 50% increased risk of hypertension [adjusted odds ratio (aOR) = 1.43, 95% confidence interval (CI): 1.01, 2.04], while no significant association was observed between SHS and hypertension. However, SHS showed a significant synergistic effect on VDD with a higher aOR of 1.79 (95% CI: 1.14, 2.80) (*P*_*interaction*_ = 0.011). This synergistic effect was more obvious when stratified by BMI (in overweight women, aOR, 95% CI =4.74, 1.65-13.60 for interaction vs 2.33, 1.01-5.38 for VDD only) and race (in Non-Hispanic Black women, aOR, 95% CI =5.11, 1.58-16.54 for interaction vs 2.69, 1.10-6.62 for VDD only).

**Conclusion:**

There exist synergistic effects of SHS and VDD on the prevalence of hypertension in American women of childbearing age, with more significant effects in women who were overweight or Non-Hispanic Black. Further studies are warranted to verify this finding in other populations, and the molecular mechanisms underlying the joint effect of SHS and VDD need to be elucidated.

**Supplementary Information:**

The online version contains supplementary material available at 10.1186/s12940-021-00803-1.

## Background

Hypertension is an important worldwide public health challenge on account of its high morbidity and the related cardiovascular risk [1]. The global prevalence related to high blood pressure (BP) has experienced a steady increase from 17.3% in 1990 to 20.5% in 2015 and is still increasing [2, 3]. Parallel to this rising global burden, the prevalence of hypertension in women of childbearing age is constantly escalating [2, 4]. Hypertension in this population is the prominent risk factor for pregnancy-related hypertension, such as preeclampsia and eclampsia, which are the leading cause of maternal and fetal mortality. It is also associated with an increased risk of childhood adverse health outcomes in the long term, including asthma [5], elevated blood pressure [6], as well as some rare childhood cancers [7]. Furthermore, postmenopausal women’s unique physiological conditions can affect BP in several hormonal ways [8], which also provides a unique risk for hypertension-related cardiovascular risk. Given the increasing prevalence and the important impacts of hypertension in women of childbearing age on gestational hypertension and postmenopausal hypertension, identification of modifiable risk factors aiming to prevent the development of hypertension is imperative.

Major risk factors of hypertension have well been identified, such as genetic predisposition, diet, physical activity, and alcohol consumption [9]. Environmental factors, such as nutrition and living habits are potentially modifiable and preventable factors. Vitamin D deficiency (VDD) is one of the most common nutritional problems all over the world, with a prevalence of about 33% in American pregnant/lactating women [10]. Although there is still inconsistency in the literature [11–13], a growing body of evidence suggests that VDD may adversely affect women’s blood pressure [14–16]. Smoking was reported to be associated with nearly 1.5-fold increased risk of hypertension [17]. Also, it may trigger vitamin D deficiency [18]. It has been reported that secondhand smoke (SHS) may have a similar effect as smoking [19]. Therefore, SHS may be involved in the relationship between VD and hypertension.

While cigarette smoking is on the decline in the US, in 2019 smoking prevalence was still 14.0% [20]. Data from a nationally representative sample suggests that up to 23.6% of females were exposed to SHS, as indicated by serum cotinine levels > 0.05 ng/mL [21]. To date, there have been several studies suggesting a significant positive association between SHS and hypertension in never smokers [19, 22, 23] including those women in childbearing age in some studies, although there existed discrepancy.

Both VDD and SHS possess characteristics related to chronic inflammation, as well as structural and functional alterations on arterial [19, 24]. In addition, exposure to SHS may precipitate VDD, which may further aggravate endothelial dysfunction, increase arterial stiffness and lead to hypertension. However, the effect of exposure to SHS with concurrent VDD on hypertension in women of childbearing age has not been evaluated till now. Taken together, we hypothesized that there may be a synergistic effect between VDD and SHS on the prevalence of hypertension in this population.

Hence, we evaluated the interaction between vitamin D levels and exposure to SHS on the prevalence of hypertension among 20-44 years old women using data from the National Health and Nutrition Examination Survey (NHANES) 2007-2014. Knowledge of this association could help health authorities in decision-making regarding policies for health promotion and intervention to prevent hypertension in women of childbearing age.

## Methods

### Study design

NHANES is conducted biennially by the Centers for Disease Control and Prevention’s National Center for Health Statistics (NCHS) since 1999, which is a cross-sectional, complex multistage, nationally representative survey that assesses population health and nutritional status that is representative of the non-institutionalized U.S. civilian population. The questionnaire data, physical examination data, and biospecimens from participants were collected. The NCHS Research Ethics Review Board reviewed and approved the study, and informed written consent was obtained from all participants before they took part in the study.

**Fig. 1:**
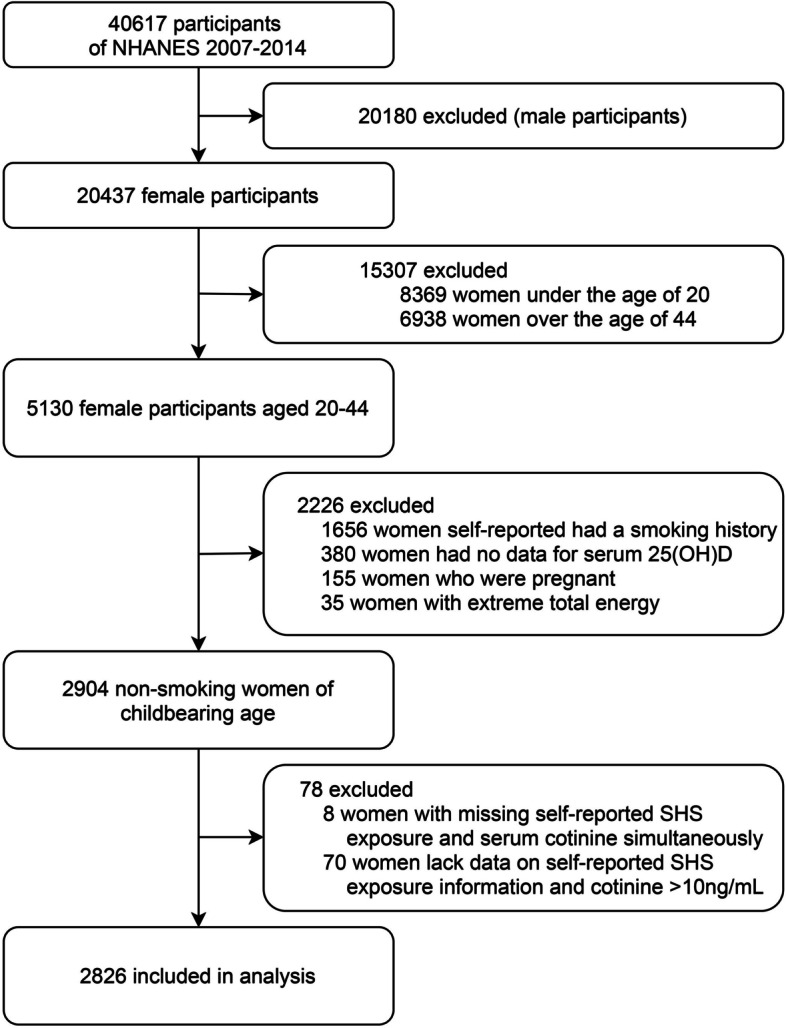
Flow chart of the screening process for the selection of eligible participants

Four consecutive survey cycles (2007-2014) with the detailed serum 25(OH)D and cotinine data were included in the present analysis (*N* = 40,617). Exclusion criteria included male participants (*n* = 20,180); female participants less than 20 years old (*n* = 8369) or older than 44 years old (*n* = 6938); self-reported a history of active smoking (*n* = 1656); missing data for 25(OH)D (*n* = 380); pregnant women (*n* = 155); abnormal energy intake (energy intake > 5000 kcal or < 500 kcal, *n* = 35); self-reported SHS exposure and serum cotinine data are simultaneously missing (*n* = 8); missing information on self-reported SHS exposure and serum cotinine > 10 ng/mL (the cut-off point for active smoking [25]) (*n* = 70). Participants chewing tobacco or using nicotine replacement therapy were also excluded because these behaviors could disturb the quantification of the biomarkers [26]. Our analysis was restricted to the 2826 women of childbearing age, defined as 20-44 years old in NHANES. The flowchart of participant inclusion is displayed in Fig. 1.

### Definition of hypertension

Blood pressure measurements were taken during the NHANES examination visits. After resting quietly in a seated position for 5 min, blood pressure was measured three times using a mercury sphygmomanometer by well-trained examiners. A fourth reading was measured if required. Then, the means of systolic blood pressure (SBP) and diastolic blood pressure (DBP) were calculated.

According to the 2017 American College of Cardiology/American Heart Association (ACC/AHA) Guideline for the Prevention, Detection, Evaluation, and Management of High Blood Pressure in Adults [9], Hypertension was defined as SBP ≥ 130 mmHg and (or) DBP ≥ 80 mmHg. At the same time, participants who answered “yes” to the question: ‘Are you now taking prescribed medicine for high BP?’ were also defined as having hypertension.

### Serum 25-hydroxyvitamin D measurement

Serum 25-hydroxyvitamin D [25(OH)D] is the widely accepted indicator of vitamin D nutritional status. Blood samples were collected from each participant at a mobile examination center (MEC) and were immediately frozen at − 30 °C for measurement of serum 25(OH)D concentrations [16]. It was measured by standardized liquid chromatography-tandem mass spectrometry (LC-MS/MS) or expressed as LC-MS/MS equivalent, depending on NHANES campaigns [27]. Vitamin D was treated as both continuous (Median and IQR, nmol/l) and categorical variable [sufficiency (≥ 75 nmol/l), insufficiency(50-75 nmol/l), and deficiency (< 50 nmol/l)] as suggested by the Institute of Medicine (IOM) [28].

### Secondhand smoke exposure

In our study, smokers were distinguished from nonusers of tobacco products through self-reporting questionnaires. Specifically, based on the answers to the questions SMQ020: “Have you smoked at least 100 cigarettes during your life?” and SMQ040: “Do you now smoke cigarettes?”, the self-reported active, former, or never smoking behavior was confirmed [26, 29].

After excluding women with a self-reported history of smoking, we categorized SHS exposure based on a combination of questionnaire and serum cotinine data. The self-reported SHS exposure was considered as present if the participants reported exposure to SHS in the last 7 days. Alternatively, participants missing responses for exposure to SHS were classified as exposed if they had serum cotinine 0.05-10 ng/mL [30–32]. Based on the previous studies, the serum cotinine cutoff value of active smoking is 10 ng/mL. Non-smoking adults who had a serum cotinine concentration ranging from 0.05 to 10 ng/mL were regarded as exposed to SHS, and those whose serum cotinine concentration less than 0.05 ng/ml were regarded as no secondhand smoke exposure. Serum cotinine was measured by an isotope-dilution high-performance liquid chromatography/atmospheric pressure chemical ionization tandem mass spectrometric (ID HPLC-APCI MS/MS) method [33].

### Covariates

We used a Directed Acyclic Graph (DAG) (www.dagitty.net/dags.html) to show the hypothesized relations between 25(OH)D, confounders, and hypertension outcomes (Supplement Fig. F1). Participants’ age, race, body mass index (BMI), physical activity and kidney disease were examined as potential confounders based on the DAG. We also carefully considered alternative DAG that included more variables, such as variables related to hypertension or 25(OH)D. As a related variable, the education level, marital status, poverty-to-income ratio (PIR), alcohol consumption and diabetes were also included.

### Statistical analysis

We tested the normality of continuous variables by Kolmogorov-Smirnov normality tests and described normal distributed variables with mean ± standard deviation, non-normal distributed variables with median (interquartile range). Student’s t-test was used to compare the mean levels between the hypertension group and the non-hypertension group if the variable was normally distributed, otherwise, the Mann-Whitney U test was adopted. Chi-square tests were used to compare the percentages of categorical variables between different groups.

For the current study, serum 25(OH)D was categorized into three groups (< 50 nmol/L, 50-75 nmol/L or ≥ 75 nmol/L) based on the criteria set by IOM, and the highest level was set as the reference category. The participants were categorized into two groups (yes or no) according to exposure to secondhand smoke, and the non-SHS was the reference category. Logistic regression models were performed to analyze the association between exposure to SHS and VDD on the prevalence of hypertension and odds ratios [OR, with 95% confidence intervals (CI)] were used to evaluate the risk associated with hypertension. In multivariate logistic regressions, model 1 adjusted for age, race, education level, marital status, PIR, and model 2 further adjusted for BMI, alcohol use, physical activity, diabetes, kidney disease. We examined interaction effects on the multiplicative scale. For multiplicative interaction, we calculated two-sided *P* values to assess the significance of each product term in the logistic regression models and compared the ORs for SHS and hypertension across strata of serum 25(OH)D. And in order to further clarify the association, the stratified analysis by BMI and race was performed to determine the joint effect of vitamin D status and exposure to SHS on hypertension in different groups. All statistical analyses were performed by using SPSS 22.0 software and *P* < 0.05 was considered statistically significant.

## Results

A total of 2826 non-smoking childbearing-age women were included in the present study. In this population, the prevalence of hypertension was 19.8%. The baseline characteristics of participants by hypertension were shown in the supplementary material (Supplement Table S1). Individuals with hypertension had a higher rate of VDD than those without hypertension (48.2% vs 41.0%, *P* = 0.008), as well as the exposure rate of secondhand smoke (39.1% vs 33.8%, *P* = 0.017). Additionally, compared with participants without hypertension, those with hypertension tended to be older, obese, had a lower educational level and a lower level of physical activity (*P* < 0.05).

Table 1 shows the effects of serum 25(OH)D and secondhand smoke on hypertension, respectively. In binary logistic regression analyses, by comparison to the vitamin D sufficient group, the OR (95% CI) of hypertension in VDD group was 1.36 (1.07, 1.74) (*P* = 0.013). In model 1, after adjusting for age, race, education level, marital status and PIR, VDD was still inversely associated with the risk of hypertension (aOR = 1.62, 95% CI: 1.19, 2.20, *P* = 0.002). Further adjusted BMI, alcohol use, physical activity, diabetes, and kidney disease in model 2, the logistic regression analysis revealed VDD as an independent predictor of hypertension (aOR = 1.43, 95% CI: 1.01, 2.04, *P* = 0.046).

**Table 1:** Odds ratios (ORs, 95% confidence intervals (CIs)) of hypertension according to serum 25(OH)D concentrations and SHS as categorical using a logistics regression model, NHANES 2007–2014(*N* = 2826)

	n (%)	Crude OR	*P-value*	Model 1 OR	*P-value*	Model 2 OR	*P-value*
**25(OH)D level**							
<50 nmol/L	270(22.5)	1.36(1.07, 1.74)**	0.013	1.62(1.19, 2.20)**	0.002	1.43(1.01, 2.04)**	0.046
50-75 nmol/L	176(17.9)	1.02(0.79, 1.33)	0.868	1.24(0.92, 1.66)	0.158	1.17(0.84, 1.63)	0.363
≥75 nmol/L	114(17.6)	Ref.	–	Ref.	–	Ref.	–
**SHS**							
Yes	219(22.3)	1.26(1.04, 1.53)**	0.018	1.14(0.91, 1.43)	0.246	1.12(0.88, 1.44)	0.364
No	341(18.5)	Ref.	–	Ref.	–	Ref.	–

Model 1: adjusted for age, race, education level, marital status, PIR

Model 2: model 1 + BMI, alcohol use, physical activity, diabetes, kidney disease

*SHS*: Secondhand smoke; **: significant at *p* <0.05

n (%): numbers and prevalence rates of hypertension of each layer

**Fig. 2:**
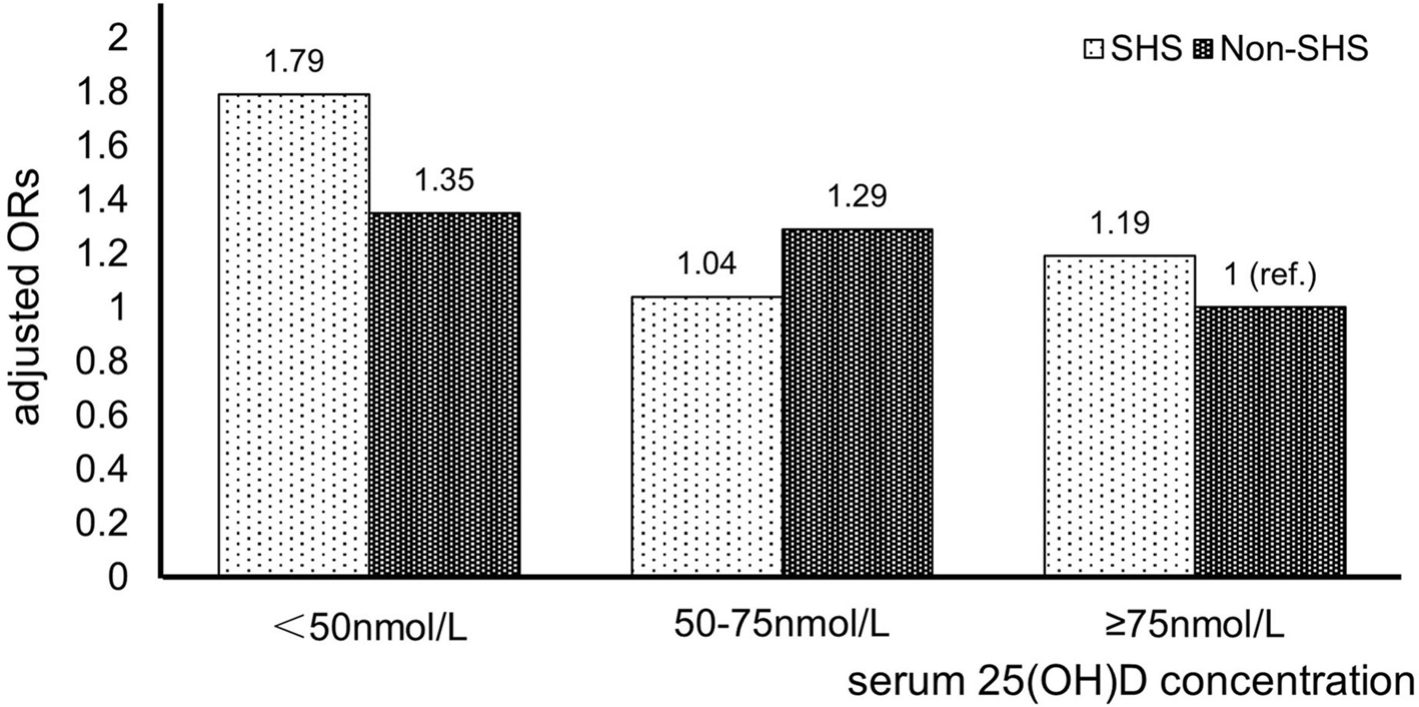
Multivariable-adjusted ORs of hypertension according to joint categories of serum 25(OH)D and SHS

The ORs with 95% CIs of hypertension based on whether exposed to SHS are shown in Table 1. Compared to those who were not exposed to SHS, those who exposed to SHS had a crude OR (95% CI) of 1.26 (1.04, 1.53) (*P* = 0.018) for hypertension. However, after adjusted according to models 1 and 2, the positive association between SHS and the risk of hypertension disappeared. Next, we divided the whole population into 6 groups according to 25(OH)D level and whether they were exposed to secondhand smoke or not (group 1: Deficiency and SHS; 2: Insufficiency and SHS; 3: Sufficiency and SHS; 4: Deficiency and non-SHS; 5: Insufficiency and non-SHS; 6: Sufficiency and non-SHS). As shown in Table 2, with sufficient 25(OH)D and no exposure to secondhand smoke (group 6) as the reference, the crude ORs with 95% CIs of hypertension for the other five groups in order were 1.79 (1.30, 2.46), 1.04 (0.71, 1.53), 1.23 (0.80, 1.90), 1.24 (0.91, 1.69), and 1.11 (0.81, 1.53), respectively. Of all the results, only VDD and exposure to SHS (group 1) had statistical significance. After adjusting the confounding factors, it was still associated with higher risk of hypertension. In Model 2, compared to those who were “Sufficiency and non-SHS”, those who were “Deficiency and SHS” had a multivariate-adjusted OR (95% CI) of 1.79(1.14, 2.80) (*P*_*interaction*_ = 0.011). The effect of the interaction between serum vitamin D status and SHS on hypertension after adjusting for confounding factors (model 2) is shown in Fig. 2.

**Table 2:** Interaction analysis of SHS and 25(OH)D on the risk of hypertension

Interaction	n (%)	Crude OR, 95% CI	*P*	Model 1 OR, 95% CI	*P*	Model 2 OR, 95% CI	*P*
Deficiency*SHS	128(26.4)	1.79(1.30, 2.46)**	0.000	2.01(1.35, 2.97)**	0.001	1.79(1.14, 2.80)**	0.011
Insufficiency*SHS	53(17.3)	1.04(0.71, 1.53)	0.840	1.17(0.76, 1.81)	0.474	1.04(0.64, 1.70)	0.863
Sufficiency*SHS	38(19.8)	1.23(0.80, 1.90)	0.347	1.25(0.77, 2.04)	0.373	1.19(0.68, 2.07)	0.538
Deficiency*non-SHS	142(19.9)	1.24(0.91, 1.69)	0.170	1.57(1.09, 2.26)**	0.015	1.35(0.89, 2.03)	0.159
Insufficiency*non-SHS	123(18.2)	1.11(0.81, 1.53)	0.504	1.39(0.98, 1.97)	0.065	1.29(0.88, 1.91)	0.197
Sufficiency*non-SHS	76(16.7)	Ref.	–	Ref.	**–**	Ref.	–

Model 1: age, race, education level, marital status, PIR

Model 2: model 1 + BMI, alcohol use, physical activity, diabetes, kidney disease

*SHS*: Secondhand smoke; ** : significant at *p* <0.05

n (%): numbers and prevalence rates of hypertension of each layer

**Fig. 3:**
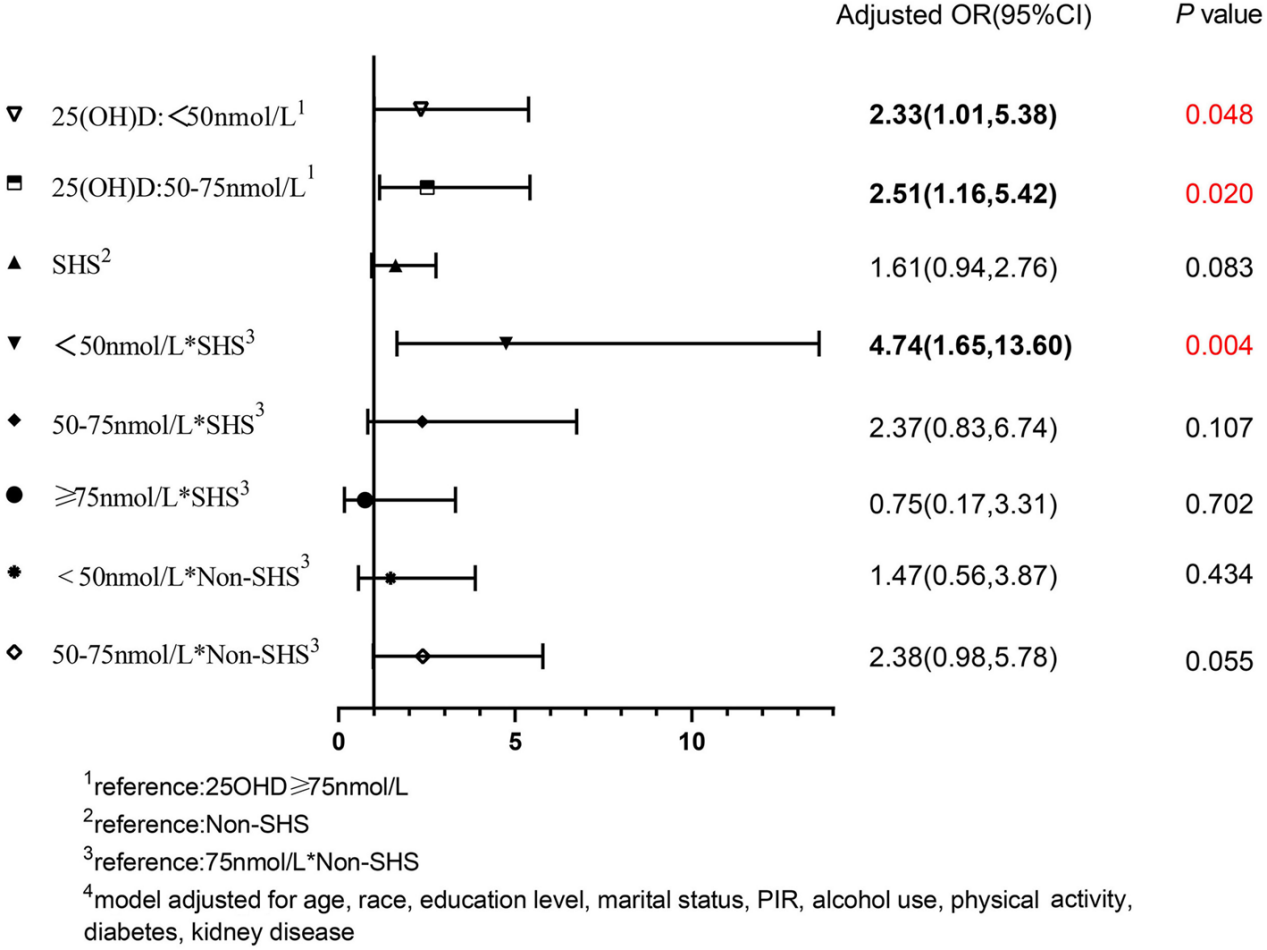
Interaction and independent effect of exposure to secondhand smoke and 25(OH) D nutrition levels on hypertension, in BMI= 25-30kg/m2 group

Stratified analyses by BMI, the association of VDD with the risk of hypertension was only significant in the overweight group (BMI = 25-30 kg/m^2^). Likewise, a significant synergistic effect between SHS and 25(OH)D was found in the overweight group only. The effects of VDD and SHS alone and their interaction on hypertension in overweight women are shown in Fig. 3. In the overweight group, the aORs with 95% CIs of hypertension were 2.33 (1.01, 5.38) and 1.61 (0.94, 2.76) in model 2 for VDD and exposure to SHS, respectively. Interaction effect analyze revealed that women in “deficiency and SHS” group has an aOR (95% CI) of 4.74(1.65, 13.60) (*P*_*interaction*_ = 0.004).

**Fig. 4:**
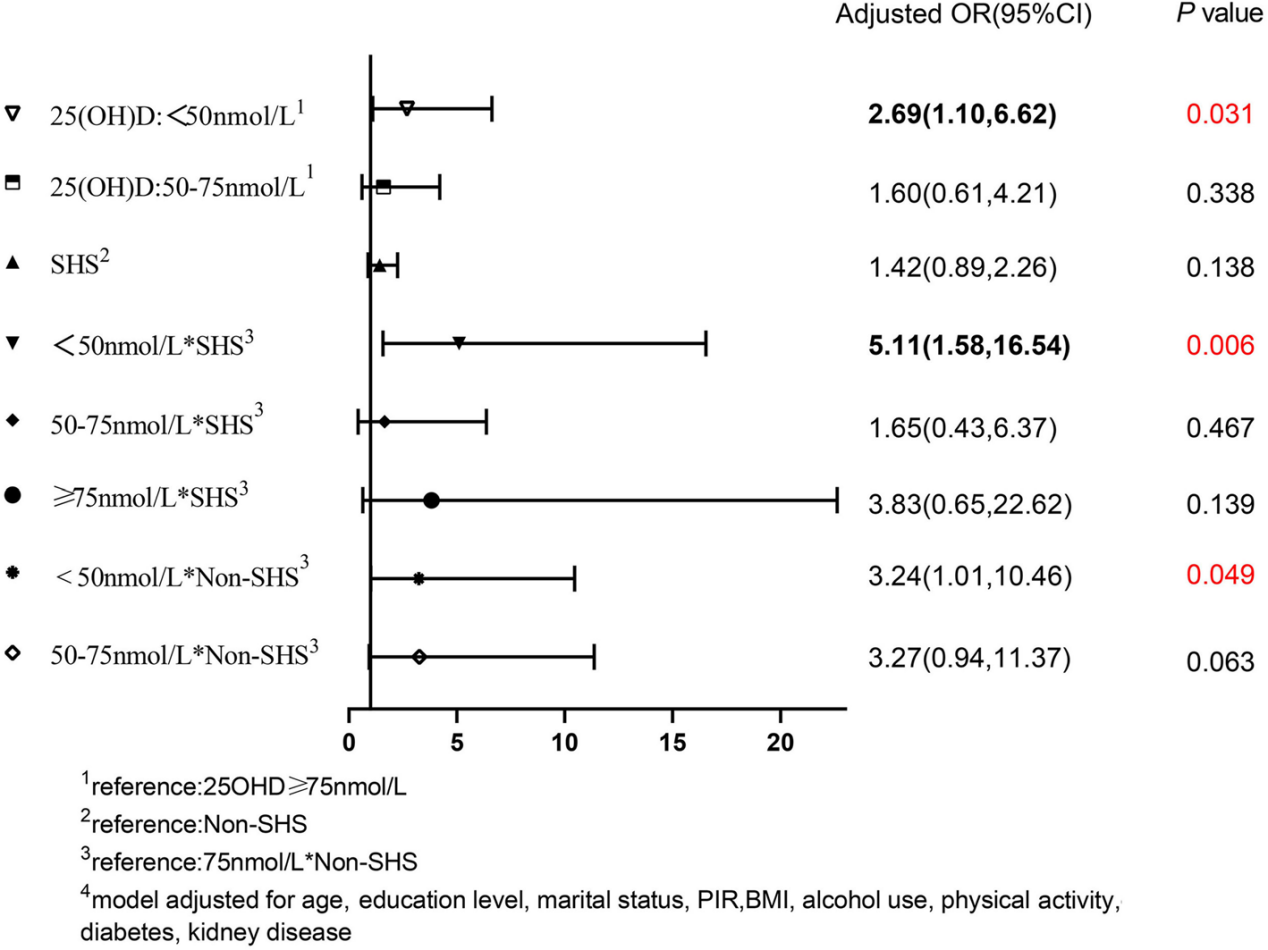
Interaction and independent effect of exposure to secondhand smoke and 25(OH)D nutrition levels on hypertension, in Race=Non-Hispanic Black group

After stratification by race, the above results were statistically significant among Non-Hispanic black. In the Non-Hispanic black stratification, compared with those with sufficient VD, the aOR (95% CI) of the effect of VDD on hypertension was 2.69 (1.10, 6.62). Compared with the “Sufficiency and non-SHS” group, the aOR (95% CI) of the “deficiency and SHS” group was 5.11 (1.58, 16.54) (*P*_*interaction*_ = 0.006). There was no significance among other races. The results are shown in Fig. 4.

## Discussion

We found a joint effect of vitamin D deficiency and exposure to secondhand smoke on the prevalence of hypertension in American women of childbearing age, and the synergistic effect was more significant in overweight or Non-Hispanic Black women.

Many studies have supported the relationship between low serum 25(OH)D levels and a high risk of hypertension. In a 14-year follow-up cohort of Caucasian women, it was suggested that vitamin D insufficiency was associated with 3 fold increased risk of systolic hypertension after adjusting for age, body fat percentage, antihypertensive medication use, and smoking [34]. Meanwhile, in the research of Forman et al., compared with the highest quartile of serum 25(OH)D, women in the lowest had an adjusted odds ratio for incident hypertension of 1.66 (95% CI: 1.11, 2.48; *P* = 0.01) [14]. Another cross-sectional study of 2098 premenopausal and 2298 postmenopausal women showed that serum 25(OH)D concentrations were associated with a lower risk of hypertension in premenopausal women [16]. However, there are still inconsistencies in the reported literature. A meta-analysis [11] included seven prospective studies of 53,375 participants showed that lower serum 25(OH)D concentrations were not associated with a greater risk of incident hypertension. Another meta-analysis, identifying 11 cohort studies and 27 RCTs in general populations with 43,320 and 3810 participants, respectively indicated that in those with serum 25(OH)D below 75 nmol/L the risk of hypertension increased substantially with decreasing vitamin D concentrations in the cohort studies, however, the supplementation with vitamin D didn’t lower blood pressure [35]. Our results are in line with the observational and experimental evidence linking lower serum 25(OH)D levels in women of childbearing age with hypertension and further provide a theoretical basis.

Till now, there has been no clear conclusion about the effects of passive smoking on hypertension in women of childbearing age, although the correlation between active smoking and hypertension has been clearly established [36, 37]. A Bulgarian study involving former smokers and nonsmokers found that passive smoking was not significantly associated with hypertension [38]. However, several studies in Asian populations reported that exposure to SHS was associated with the risk of high blood pressure. In rural Chinese nonsmoking female aged 33 ~ 82, passive smoking was found to be associated with a higher risk of hypertension [39]. Another prospective cohort study in 106,268 Korean evaluated the association between exposure to SHS in self-reported never-smokers verified by urinary cotinine and hypertension [40], and they discovered that current SHS exposure at home [1.22 (1.11, 1.33)], as well as current SHS exposure only at the workplace [1.15(1.02, 1.29)], significantly increased risk of hypertension. Similar relationships were reported in children and adolescents. The study of the effects of SHS on blood pressure of 3579 children and adolescents aged 8 to 17 using NHANES showed that children exposed to high levels of SHS were 1.97 (95% CI: 1.31, 2.95) times more likely to be in the range of hypertension than those without SHS exposure [41]. A large-scale study, conducted among 42,745 children ages 7-18 years, found that parental self-report of household smoking was associated with 11% higher risk of childhood hypertension in girls (aOR = 1.11, 95% CI: 1.02, 1.20, [42]. No significant associations were found in boys. Similarly, among adults (≥ 18 years) in the United States of America, SHS was associated with increased odds of hypertension among women (aOR = 1.24, 95% CI: 1.24, 1.24) but not among men. However, SHS exposure of the participants was assessed only by the self-reported questionnaire in these studies.

In our study, exposure to SHS was associated with greater hypertension risk. However, the association became non-significant after adjusting for the potential confounders. The results were contrary to our expectations; it may be due to the research design, the selection of the population, and the different criteria for judging secondhand smoke exposure. Further research is worth being conducted on the hypothesis that exposure to secondhand smoke increases the risk of hypertension in women of childbearing age.

Our epidemiological data indicate that SHS represents an important environmental factor contributing to hypertension in females with VDD. The possible underlying mechanisms are as follows. On one hand, studies have shown that smoking may trigger relevant potential pathways disrupting VD endocrine system (VDES) leading to VDD. First, smoking inhibits the expression of CYP27B1(the key enzyme required for activation of VD) [43], decreases the level of serum parathyroid hormone (PTH), increases the exposure to cadmium and lead, and increases the expression of CYP24A1 (the key enzyme required for 24-hydroxylase activity) to reduce the levels of 25(OH)D and 25(OH)_2_D in serum. Second, smoking inhibits the intake of VD from diet [18]. Third, smoking promotes skin aging and thus hinders the synthesis of VD [18]. On the other hand, the effect of the ability of vitamin D to negatively regulate the renin-angiotensin system (RAS) and the association with endothelial vasodilator dysfunction [44–46]. Passive smoking may have adverse effects on vasoconstriction and/or vasodilation, due to nicotine causes vasoconstriction, which results in transient increases in BP. Further, Passive smoking may also lead to vascular endothelial dysfunction by affecting vascular endothelial cells [47, 48]. Zhang J et al. [49] posited the endothelial dysfunction was mediated as a result of systemic inflammation. The inflammatory responses and oxidative stress responses due to vitamin D deficiency may be aggravated by SHS. This may aggravate the effect on vascular endothelial function and lead to hypertension when both vitamin D deficiency and secondhand smoke exposure are present. Our results are consistent with several previous studies evaluating the independent associations between vitamin D and hypertension, and our identification of the synergistic effect of secondhand smoke exposure may support the hypothesized biological mechanisms of these associations.

Overweight enhances the synergistic associations between VDD and SHS on hypertension, which has potential clinical and public health relevance. Potential pathways may involve the effects of overweight and SHS exposure on the interdependence of airway and systemic oxidative stress and inflammation [50], as well as their effects on VD deficiency [51]. These findings offer new insight into why hypertension in overweight and obese women may be more severe. However, we did not find the interaction in the obesity group. Our conjecture remains to be further confirmed.

There are several strengths in our study. First, hypertension was defined by using the new diagnostic criteria for hypertension in 2017. The new guidelines lower the standard of high blood pressure and will require more adults to receive anti-hypertensive treatment. And it can prevent about 610,000 CVD events and 334,000 deaths each year in the United States alone [52], reducing the resulting economic burden. Second, to our knowledge, this is the first study to explore the associations between the combined exposure of vitamin D deficiency and SHS and the risk of hypertension among the general American women of childbearing age, which has significant public health relevance. Third, we used the SHS exposure self-reported questionnaire and the objective index of serum cotinine simultaneously to classify whether exposed to SHS or not, which made the result more reliable. Previous studies may be limited by exposure assessment because self-report of exposure to SHS may not be as accurate as biological markers of exposure. The use of biomarkers could reduce measurement error. Cotinine is the principal metabolite of nicotine and can be measured in serum, urine, or saliva; it has been effectively used as a sensitive biomarker for exposure to tobacco in both active and SHS due to its long half-life [53].

However, there are still several limitations in our study. First, because of the cross-sectional design of the study, it is difficult to determine the causality. Second, according to the information of the questionnaire, women who have self-reported active smoking were excluded, and this method is often imprecise because of conscious or unconscious mis-recordings and under-reporting and there might be ineluctable recall bias. Third, we cannot rule out all the possible residual confounding from unmeasured confounders.

## Conclusion

In summary, vitamin D deficiency was associated with a higher risk of hypertension and there exist synergistic effects of secondhand smoke exposure and VDD on the prevalence of hypertension in American women of childbearing age, with more significant effects in women who were overweight or Non-Hispanic Black. These findings add new theoretical support for the potential risk of hypertension and reinforce the view that low 25(OH)D and secondhand smoke exposure are risk factors for hypertension. And these could help facilitate planning and guiding targeted strategies, which may include a total ban or more stringent restrictions on smoking, as well as population VD supplements to prevent and control high blood pressure in women of childbearing age around the world. In addition, our study highlights the need for future prospective studies and mechanism research to further explore the burden and impact of secondhand smoke exposure and vitamin D deficiency on the outcomes of women of childbearing age or pregnant women and fetuses.

## Supplementary Information

**Additional file 1 **The characteristics of study population by hypertension, NHANES 2007-2014 (*N* = 2826) is shown in Supplement Table S1. The DAG is shown in Supplement Fig. F1. **Supplement Table S1**: Characteristics of study population by hypertension, NHANES 2007-2014 (*n* = 2826). **Supplement Fig. F1**: Directed Acyclic Graphs for the Causal Effect of 25(OH)D or the interaction with SHS on hypertension.

## Data Availability

Data are available at NHANES - National Health and Nutrition Examination Survey Homepage (cdc.gov). Further database is available on request.
