# Supplementary material for: Joint effect of 25-hydroxyvitamin D and secondhand smoke exposure on hypertension in non-smoking women of childbearing age: NHANES 2007-2014
Source: Environ Health. 2021 Nov 15;20:117. doi: 10.1186/s12940-021-00803-1 (PMC8591921; doi:10.1186/s12940-021-00803-1)
Supplement: Supplementary file 1 — Additional file 1 The characteristics of study population by hypertension, NHANES 2007-2014 (N = 2826) is shown in Supplement Table S1. The DAG is shown in Supplement Fig. F1. Supplement Table S1: Characteristics of study population by hypertension, NHANES 2007-2014 (n = 2826). Supplement Fig. F1: Directed Acyclic Graphs for the Causal Effect of 25(OH)D or the interaction with SHS on hypertension. [file 12940_2021_803_MOESM1_ESM.docx]

Supplement table S1: Characteristics of study population by hypertension, NHANES 2007-2014 (n=2826)

| Characteristics | Total | Hypertension | Non-hypertension | *P*-value |
| --- | --- | --- | --- | --- |
| n (%) | 2826 | 560 (19.8) | 2266(80.2) |  |
| Age, Median (IQR)  Race (%)  Mexican America  Non-Hispanic White  Non-Hispanic Black  Others  Education level (%)  Below high school  High school  Above high school  Marital status (%)  Never married  Married/living with partner  Separated/widowed/divorced  Family Poverty Index Ratio (%)  PIR＜1  PIR≥1 | 32(26,39)  596(21.1)  893(31.6)  575(20.3)  762(26.9)  499(17.7)  473(16.8)  1851(65.6)  879(31.1)  1667(59.0)  280(9.9)  651(24.8)  1974(75.2) | 37(31,41)  89(15.9)  178(31.8)  183(32.7)  110(19.6)  103(18.4)  115(20.6)  341(61.0)  149(26.6)  331(59.1)  80(14.3)  144(28.1)  369(71.9) | 31(25,38)  507(22.4)  715(31.6)  392(17.3)  652(28.8)  396(17.5)  358(15.8)  1510(66.7)  730(32.2)  1336(59.0)  200(8.8)  507(24.0)  1605(76.0) | **＜0.001**  **＜0.001**  **0.014**  **＜0.001**  0.056 |
| BMI, kg/m^2^, Median (IQR)  < 25 kg/m^2^  25 to < 30 kg/m^2^  ≥ 30 kg/m^2^  SBP, mmHg, Median (IQR)  DBP, mmHg, Median (IQR)  25(OH)D_2_+25(OH)D_3_, nmol/L,  Median (IQR)  25(OH)D_2_+25(OH)D_3_(%)  < 50nmol/L  50-75nmol/L  ≥ 75nmol/L  25(OH)D_3_ nmol/L, Median (IQR)  Serum cotinine, ng/mL,  Median (IQR)  Secondhand smoke exposure (%)  Total energy, kcal/d, Median (IQR)  Alcohol drinking (%) | 26.8(22.8,32.4)  1098(39.2)  717(25.6)  987(35.2)  110(103,117)  68(62,75)  55.1(39.3,72.8)  1198(42.4)  981(34.7)  647(22.9)  52.2(36.7,69.8)  0.024(0.011,0.074)  984(34.8)  1806(1377,2316)  1505(61.7) | 31.6(26.5,39.2)  106(19.2)  118(21.3)  329(59.5)  123(117,133)  81(73,85)  51.8(36.5,69.9)  270(48.2)  176 (31.4)  114(20.4)  47.9(33.5,66.6)  0.028(0.011,0.098)  219(39.1)  1806(1394,2263)  286(57.2) | 25.9(22.3,31.0)  992(44.1)  599(26.6)  658(29.3)  107(102,113)  67(61,72)  55.8(39.8,73.5)  928(41.0)  805(35.5)  533(23.5)  53.0(37.7,70.5)  0.023(0.011,0.069)  765(33.8)  1806(1375,2322)  1219(62.9) | **＜0.001**  **＜0.001**  **＜0.001**  **0.003**  **0.008**  **＜0.001**  **0.007**  **0.017**  0.855  **0.019** |
| Work Physical activity (%)  Vigorous  Moderate  Other  Recreational Physical activity (%)  Vigorous  Moderate  Other  Diabetes (%)  Kidney disease (%)  PIR, Poverty Index Ratio; BMI, body mass index; SBP, systolic blood pressure; DBP, diastolic blood pressure | 317(11.2)  665(23.5)  1844(65.3)  796(28.2)  755(26.7)  1275(45.1)  85(3.0)  43(1.5) | 66(11.8)  127(22.7)  367(65.5)  117(20.9)  169(30.2)  274(48.9)  53(9.5)  23(4.1) | 251(11.1)  538(23.7)  1477(65.2)  679(30.0)  586(25.9)  1001(44.2)  32(1.4)  20(0.9) | 0.808  **＜0.001**  **＜0.001**  **＜0.001** |

Supplement figure F1
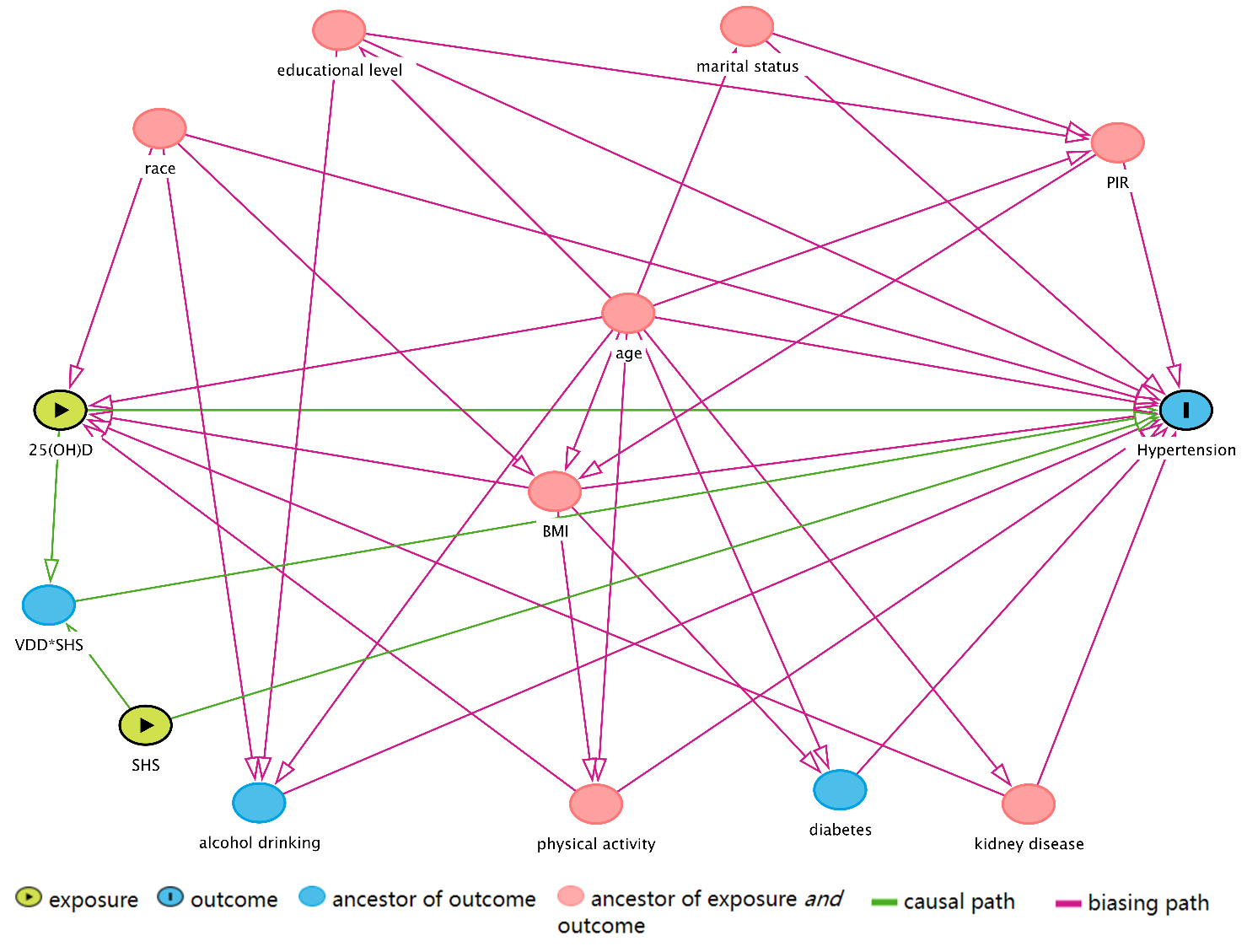
: Directed Acyclic Graphs for the Causal Effect of 25(OH)D or the interaction with SHS on hypertension
